# Reducing the harms of cannabis use in youth post-legalization: insights from Ontario youth, parents, and service providers

**DOI:** 10.1186/s12954-024-01112-9

**Published:** 2024-11-06

**Authors:** Toula Kourgiantakis, Angie Hamilton, Christine Tait, A. Kumsal Tekirdag Kosar, Carrie K. Y. Lau, Sandra McNeil, Eunjung Lee, Shelley Craig, Abby L. Goldstein

**Affiliations:** 1 École de travail social et de criminologie, Université Laval, Québec, QC G1V 0A6 Canada; 2 Families for Addiction Recovery (FAR), Toronto, ON Canada; 3 Factor-Inwentash Faculty of Social Work, University of Toronto, Toronto, ON Canada; 4 Department of Applied Psychology and Human Development, Ontario Institute for Studies in Education, Toronto, ON Canada

**Keywords:** Youth cannabis use, Parents, Family, Service providers, Canada, Cannabis legalization, Lower-risk cannabis use guidelines, Harm reduction, Public health

## Abstract

**Background:**

Canada has one of the highest prevalence of cannabis use globally, particularly among young adults aged 20–24 (50%) and youth aged 16–19 (37%). In 2018, Canada legalized recreational cannabis with the aim of protecting youth by restricting their access and raising public awareness of health risks. However, there has been limited qualitative research on the perceptions of harms associated with youth cannabis use since legalization, which is crucial for developing effective harm reduction strategies. This qualitative study examined perceptions of cannabis use among youth from the perspectives of youth, parents, and service providers. We explored how participants described the perceived risks or harms associated with youth cannabis use, as well as how they described their own and others’ approaches to reducing cannabis-related risks and harms.

**Methods:**

This qualitative study used a community-based participatory research approach in partnership with Families for Addiction Recovery (FAR), a national charity founded by parents of youth and young adults with addiction issues. Virtual semi-structured interviews were conducted, and the data were analyzed using thematic analysis.

**Results:**

The study included 88 participants from three key groups (*n* = 31 youth, *n* = 26 parents, *n* = 31 service providers). Two main themes emerged regarding perceived risks or harms associated with cannabis use: (1) concerns about cannabis-related risks and harms, including addiction, brain development, impact on family, and various adverse effects on areas such as motivation, concentration, finances, employment, education, physical and mental health; and (2) minimization of risks and harms, featuring conflicting messages, normalization, and perceptions of cannabis being less harmful than other substances. Additionally, two themes related to harm reduction approaches were identified: (1) implementation of harm reduction, and (2) challenges in implementing a harm reduction approach. Specific challenges for each participant group were noted, along with structural barriers such as unavailable and inaccessible services, easy access to cannabis, inadequate public education, and insufficient information on lower-risk cannabis use guidelines.

**Conclusions:**

Youth cannabis use is a significant public health concern that requires a multi-pronged approach. Developing youth-centered harm reduction strategies that recognize the developmental needs and vulnerabilities of youth, as well as the important role of families, is imperative.

Canada has the highest cannabis use prevalence worldwide [1], particularly among youth and young adults. Among young adults aged 20 − 24, 51% report using cannabis in the past year, followed by youth aged 16 − 19 at 37%, compared to 21% of adults over the age of 25 [2]. The Canadian Cannabis Survey (CCS) shows higher cannabis use among sexual and gender minority young adults, with 66% of bisexual and 58.3% of gay or lesbian individuals reporting use in the past month, compared to 45% of their heterosexual peers. When the Government of Canada legalized recreational cannabis in 2018, the Cannabis Act declared that one of its primary objectives was public health and safety, with a specific focus on protecting young people by reducing their access to cannabis. The Canadian Paediatric Society (CPS) raised concerns about the impact of legalization on youth and young adults under the age of 25 who are considered one of the most vulnerable age groups because their brains are not yet fully developed [3].

As Canada prepared for legalization, the government formed a Task Force to develop a framework for cannabis regulation, promoting a public health approach. The Task Force acknowledged the risks to youth and young adults under the age of 25, recommending “a public health approach that aims to delay the age of initiation, reduce the frequency of use, reduce higher-risk use, reduce problematic use and dependence, expand access to treatment and prevention programs, and ensure early and sustained public education and awareness” [4, p. 15].

Cannabis use in youth is a serious public health concern associated with numerous adverse effects, including cognitive impairments [5, 6], depression [5, 7–9], anxiety [9, 10], bipolar disorder [11], and suicidality [11–13]. Cannabis use is also linked to psychosis, with substantial evidence indicating that early onset of cannabis use increases the risk or severity of psychosis outcomes, prompting recommendations to avoid cannabis use during adolescence or to delay initiation as much as possible [14]. The risks of psychosis are linked to higher levels of tetrahydrocannabinol (THC) in cannabis products. Since the 1980s, the THC content has increased from less than 5% to 25%, with cannabis concentrate products available in many jurisdictions containing 60 to 90% THC [3]. The Canadian Paediatric Society has recommended that the government limit the concentration of THC in products legally sold to young adults aged 18–25 [3]. Additionally, regarding awareness of THC levels, less than one-third of youth and young adults are knowledgeable about THC levels in consumed products [2, 15].

Since legalization, the rates of cannabis use, and cannabis use disorder have increased among young adults aged 18– 24 [16–21]. For youth under the age of 18, many studies show that the rates have stayed the same [20, 22, 23]. However, Nguyen et al. [22] examined cannabis use in 15- to 18-year-olds and found that while there was not a significant increase in its prevalence, there were increases in the initiation of cannabis use among never users and easier access to cannabis since legalization.

Furthermore, the rates of emergency department visits for cannabis-related intentional and unintentional injuries have increased since legalization for youth under 18, as well as young adults 18–25 [24–32]. Several studies have linked the rise in emergency departments visits to the commercialization of cannabis, which includes promotion, marketing, and widespread access to retail stores [28–30, 33]. Youth report that access to cannabis is very easy since legalization, even for youth under the minimum legal age [19, 34, 35].

To reduce the risks associated with cannabis use, a group of international experts developed the Lower Risk Cannabis Use Guidelines (LRCUG) as a public health prevention and intervention tool [36–38]. These guidelines are supported by government agencies and health organizations to mitigate cannabis-related risks and harms. However, research specifically focused on lower-risk cannabis use guidelines for youth, as well as guidelines for other substances tailored to youth, has been limited [39]. To better understand guidelines for lower-risk substance use among youth, Moebes et al. [40] examined what has been published, the type of information provided, how guidelines differ by substance, and how they are tailored specifically for youth. The authors describe lower-risk substance use guidelines (LRSUGs) as “tools that provide information regarding evidence-based harm reduction strategies that can help people who use drugs navigate the risks associated with substance use” [40, p. 2]. The review identified inconsistencies in the accuracy and reliability of LRSUG information disseminated by various organizations, noting that fewer than half of these guidelines are based on robust scientific evidence and sources. The review also highlighted significant variability in cannabis use guidelines, particularly concerning abstinence, dosage, and frequency of use. Furthermore, the authors criticized the one-size-fits-all approach of harm reduction guidelines, which fails to consider the age-specific needs and characteristics of different population groups. Another significant finding of the review was that less than half of the LRSUGs discussed available treatment and care options, which they underlined is a “clear missed opportunity for facilitating treatment and care” [40, p. 9].

Given the importance of LRCUGs in addressing cannabis use harms, it is important to consider public awareness and education surrounding these risks. Public awareness of the risks associated with cannabis use in youth is low. According to the Canadian Cannabis Survey (CCS), only 14.8% of individuals over 16 who regularly smoked cannabis perceived it as a serious risk, compared to 75.7% of those who regularly smoked tobacco over the past 12 months [2]. One of the purposes of the Cannabis Act is to “enhance public awareness of the health risks associated with cannabis use” [41, pp. 6–7]. However, a report by the Canadian Centre on Substance Use and Addiction (CCSA) highlighted that cannabis literacy is inadequate, underlining misinformation about cannabis and the need for more public education, particularly for priority populations such as youth [42]. A study by Bishop et al. [43] found that youth expressed a need for cannabis-informed education and harm-reduction approaches to minimize harms to oneself and be better able to support others using cannabis.

Harm reduction, an approach that emerged in the 1990s, aims to reduce or mitigate the harms associated with substance use. It can be applied at the individual (micro), community (meso), or societal (macro) levels [44]. The philosophy of harm reduction is for individuals to “come as they are” [45 p788] with “a pragmatic yet compassionate set of strategies” [45, p. 789]. Harm reduction seeks to minimize the adverse consequences of substance use, encompassing a continuum of options to minimize harm one step at a time [44, 46]. Marlatt [45] outlined four central assumptions, principles, and values for harm reduction: (1) Harm reduction is a public health alternative to the disease, criminal, and moral model of substance use; (2) Harm reduction uses a continuum approach to reducing harm with a range of options from extremely harmful to less harmful consequences. Any movement in the direction of reducing harm is viewed as a step in the right direction, and the continuum includes abstinence; (3) Harm reduction is a bottom-up approach that is grounded in service user advocacy; and (4) Harm reduction promotes low barrier access to services that reduce stigma and involve outreach and partnerships with community. Adinoff & Cooper [47] explain that a true harm reduction model aims to find a balance between the extremes of cannabis prohibition and unregulated legalization through strict regulation. Researchers and practitioners argue that we need more youth-centered harm reduction strategies and LRCUG to inform policies, practice, and research [40, 48, 49].

While research on mitigating the harms of cannabis use among youth is growing, numerous gaps remain in the existing literature. A recent scoping review of youth cannabis use literature since legalization in Canada found that 92% of studies are quantitative, and 68% use secondary data [18]. Qualitative or mixed method studies, that examine in greater depth, the experiences and perspectives of those most affected are limited. This qualitative study aimed to fill some of these gaps by exploring perceptions of youth cannabis use, from the perspectives of three key participant groups: youth, parents, and service providers. Specifically, we examined two primary research questions: (1) How do participants describe the perceived risks or harms associated with youth cannabis use? (2) How do participants describe their own and others’ approaches to reducing cannabis-related risks and harms?

## Methods

### Design and setting

This study was conducted using a descriptive qualitative research design, which is suitable for topics that have received limited exploration [50]. Additionally, we used a community-based participatory research approach, driven by community needs and strengths [51, 52]. The study involved a collaborative partnership with Families for Addiction Recovery (FAR), a Canadian charity founded by parents of youth with substance use concerns. FAR provides support to parents and caregivers through free, individualized, or group peer support. FAR also engages in research, education, and advocacy, focusing on the need for accessible, compassionate, evidence-based treatment on demand, protective health laws, protective drug policies and the elimination of stigma [53]. The Research Ethics Board at the University of Toronto granted ethics approval for this study (#42006).

### Participant sample and recruitment

The sampling strategy used in this study was purposeful sampling [54]. We included youth, parents, and service providers in Ontario who could contribute to our understanding of youth cannabis use through their professional and/or lived experiences. Specifically, our inclusion criteria were as follows: (1) youth aged 16–24 regularly who used cannabis regularly (at least once per week), (2) parents/caregivers of youth aged 16–24 who used cannabis regularly (at least once per week), and (3) service providers providing mental health related services to youth aged 16–24 in a mental health and/or addiction related setting. It is important to note that we were not specifically recruiting parent and youth participants from the same family for this study, although this was not an exclusion criterion either. The selection of the 16–24 age group was intentional, recognizing this phase as a critical transition stage between childhood and adulthood, and a period where most substance use and mental health concerns typically emerge [55]. Recent studies also show that this age group has the highest rates of cannabis use in Canada [18]. For simplicity, we use the term “youth” to refer to participants under 18, as well as those aged 18–24 who took part in the study, including those described by the parent and service provider groups. When citing other studies in different sections of the paper, we use the same terms as the authors.

Participants from all groups were recruited through social media, mental health agencies, clinics, hospitals, professional associations, and student associations. Parents were additionally recruited through FAR. The recruitment flyer included a link to the study information and consent form hosted on Qualtrics. After reviewing the form and providing electronic consent, participants were contacted by a research assistant (RA) to schedule a virtual interview. To enhance accessibility, participants were also given the option to call, text, or email the RA for more information about the study before consenting electronically. Following the interview, participants received an honorarium in the form of a $30 gift card as a token of appreciation for their time.

### Data collection

The research team collected data from March 7, 2022, to October 17, 2022, through an online sociodemographic questionnaire and semi-structured individual interviews. Each interview was approximately 60 min long and conducted via Zoom. The interviews were facilitated by the Principal Investigator (TK) and one of the RAs (CT, CL, KTK, SM). After providing electronic consent and prior to the interview, participants completed a demographic questionnaire.

The youth demographic survey consisted of 31 questions covering characteristics such as age, region of Ontario, education, employment, gender, sexual orientation, race, religion, and living arrangements. Additionally, it included questions about cannabis use, use of other substances, behavioral addictions, mental health, and services received for mental health and addiction-related concerns. The parent and caregiver demographic survey, consisted of 33 questions with similar inquiries about age, region of Ontario, education, employment, gender, sexual orientation, race, and religion. Furthermore, the parent questionnaire explored the parent’s mental health, as well as their perceptions of their youth’s mental health, substance use, and services accessed. Likewise, the service provider survey, comprising 29 questions, covered demographic information, along with questions about the profession, area of specialization, and years of experience. Before implementation, the demographic surveys, consent forms, and contact forms were pilot tested by research team members.

A semi-structured interview guide was developed for each group (youth, parent, and service provider) and reviewed by the research team. The youth guide comprised 15 open-ended questions regarding their perceptions of their cannabis use, legalization, other substance use, service access, COVID-19 pandemic impacts, stigma, discrimination, racism, and recommendations for improving policies, services, education, and training. The parent guide contained 16 open-ended questions regarding their youth’s cannabis use, legalization, other substance use, COVID-19 pandemic impacts, communication with their youth, stigma, discrimination, racism, and recommendations. Similarly, the service provider guide consisted of 20 open-ended questions concerning their workplace setting, role, youth cannabis use, legalization, youth using other substances, personal experience with cannabis, opinions on the treatment of mental health and substance use, service description and approaches with youth, COVID-19 pandemic impacts, stigma, discrimination, racism, and recommendations. The interviews were audio-recorded and transcribed. The transcriptions were de-identified by assigning an ID code.

### Data analysis

For data analysis, we employed Braun & Clarke’s [56] six stages of thematic analysis to identify themes and patterns within the data. These stages include: (1) data familiarization; (2) generating initial codes; (3) generating initial themes from coded data; (4) reviewing themes; (5) defining and naming themes; and (6) interpreting and reporting. We used Dedoose, an online software, to organize, synthesize, and code the data. The analysis was completed by the Principal Investigator (PI) and three research assistants (RAs) (CT, CL, KTK).

During the initial phase, research team members familiarized themselves with the data by reading transcripts, writing memos, and creating a codebook detailing code descriptions with exemplars. Initial codes emerged during this review and were identified in the second stage. Each transcript had a first and second coder, with the PI reviewing each double-coded transcript to ensure consensus and resolve discrepancies. Discrepancies were addressed in weekly meetings, and the codebook was updated as coding evolved. Once coding was completed, we reviewed the codes and their excerpts to identify overarching themes, which were discussed with other research team members, including our community partner.

Rigor and trustworthiness were enhanced through credibility, dependability, confirmability, and transferability [57]. To enhance credibility, we used triangulation of data sources with three participant groups, and researcher triangulation which involved always having two independent coders and a third resolving discrepancies. Our research team was diverse, including researchers, service providers, a community partner, and students with diverse backgrounds in terms of race, ethnicity, religion, gender, ability, and age. We also included team members with extensive clinical experience in community mental health, and those with lived experiences. An audit trail increased dependability and confirmability through detailed notes and regular meetings from the start of the research project, throughout data collection, and again during data analysis. Prolonged engagement with participants and data enhanced credibility, dependability, and confirmability. Thick description of participant stories, experiences, and quotes, enhanced transferability. Finally, trustworthiness was strengthened through reflexivity on the part of the research team members. We examined how our conceptual frameworks, training, diverse identities, experiences, values, and assumptions affected research decisions in all phases of the study. To minimize bias, research team members wrote reflexive memos during analysis, regularly debriefed throughout the coding process, and kept detailed notes of all meetings.

## Results

The sample included a total of 88 participants (*n* = 31 youth, *n* = 26 parents, *n* = 31 service providers). Demographic information for all three groups is presented in Table 1, though some details could not be included. For example, more than half (58%) of the youth participants lived with their family, 29% lived on their own, 6% in shelters, 3% in a treatment center, and 3% in foster care.

**Table 1:** Self-reported participant characteristics (*N = 88*)

Characteristics *n* (%)	Youth (*n* = 31)	Parents (*n* = 26)	Service Providers (*n* = 31)
Age of youth			
16–18	6 (19%)	–	–
19–20	9 (29%)	–	–
21–24	16 (52%)	–	–
Age of parents & service providers			
20–29	–	–	8 (26%)
30–39	–	–	11 (35%)
40–49	–	5 (19%)	7 (23%)
50–59	–	17 (65%)	4 (13%)
60–69	–	4 (16%)	1 (3%)
Gender			
Woman	17 (55%)	18 (69%)	23 (74%)
Man	5 (16%)	8 (31%)	7 (23%)
Gender diverse (non-binary, trans)	9 (29%)	–	1 (3%)
Sexual orientation			
Straight/heterosexual	9 (29%)	22 (84%)	19 (63%)
Lesbian/Gay/Bisexual/Queer/Pansexual	16 (52%)	3 (12%)	9 (29%)
Not sure or questioning	–	–	2 (6%)
Multiple selected	6 (19%)	–	–
None of these identities	–	1 (4%)	1 (3%)
Race			
Black	1 (3%)	–	3 (10%)
Indigenous	–	1 (4%)	–
South Asian	5 (16%)	–	2 (6%)
East/Southeast Asian	3 (10%)	1 (4%)	2 (6%)
Latino/Latina/Latinx	1 (3%)	1 (4%)	–
Mixed race	1 (3%)	3 (12%)	3 (10%)
Another race category	2 (6%)	–	1 (3%)
White	18 (59%)	20 (76%)	20 (65%)
Religion			
Christian	4 (13%)	17 (65%)	9 (29%)
Jewish	1 (3%)	–	4 (13%)
Muslim	3 (10%)	1 (4%)	1 (3%)
Sikh	1 (3%)	–	–
Hindu	1 (3%)	–	–
Indigenous spirituality	–	–	1 (3%)
Multiple religions	1 (3%)	–	–
No religion	18 (59%)	6 (23%)	13 (43%)
Other religion or spirituality	2 (6%)	2 (8%)	3 (9%)
Enrolled in school (youth)			
Yes	16 (52%)	–	–
No	15 (48%)	–	–
Employment			
Full-time	9 (29%)	14 (54%)	–
Part-time	9 (29%)	5 (19%)	–
Retired	–	4 (15%)	–
Homecare	–	1 (4%)	–
Unable to work	2 (6%)	1 (4%)	–
Unemployed	11 (36%)	1 (4%)	–
Region			
Central Ontario	21 (69%)	15 (58%)	21 (67%)
Eastern Ontario	3 (10%)	7 (27%)	3 (10%)
Southwestern Ontario	2 (6%)	4 (15%)	3 (10%)
Northeastern & Northwestern Ontario	3 (9%)	–	3 (10%)
Multiple regions selected	2 (6%)	–	1 (3%)

Almost two-thirds of parent participants had concerns about their sons’ cannabis use (65%), 31% had concerns about their daughters’ cannabis use, and one had concerns about their trans son’s cannabis use. More than half (58%) of the parents reported that their youth was living at home, 15% reported their youth was living on their own, 8% reported their youth was away at school, 4% in a treatment center, and 15% in other living arrangements. Although we did not specifically state that parents or youth were ineligible if they had a family member participating in the study, the two samples were independent, and we are not aware of any youth and parent participants being from the same families.

The service providers included social workers (55%), physicians (24%), psychotherapists (6%), nurses (6%), child & youth workers (3%), addiction counsellors (3%), and community support workers (3%). About 58% had 10 years or less of experience in mental health and addictions, while the remaining 42% had between 11 and 21 + years of experience. Table 2 provides information about the mental health and substance use of the youth participants while Table 3 outlines the mental health and substance use of the parent participants, including details about the youth they support. The next section presents the results for each research question, supported by participant quotes. We use the following notations to identify the sources of quotations: ‘Y’ for youth, ‘P’ for parent, and ‘SP’ for service provider.

**Table 2:** Mental health & substance use of youth participants (n = 31)

Mental health and substance use of youth	n (%)
Youth has concerns about their mental health	
Yes	21 (68%)
No	6 (19%)
Prefer not to say	4 (13%)
Youth has a diagnosed mental health concern	
Yes	16 (52%)
No	15 (48%)
Youth is using substances other than cannabis (including alcohol)	
Yes	13 (42%)
No	18 (58%)
Youth is concerned about their cannabis use	
Yes	17 (55%)
No	10 (32%)
Unsure	4 (13%)
Others have concerns about youth’s cannabis use	
Yes	17 (55%)
No	10 (32%)
Missing data	4 (13%)
Frequency of cannabis use	
1–2 times per week	10 (32%)
4–6 times per week	4 (13%)
Once per day	7 (23%)
Multiple times per day	10 (32%)
Modes of cannabis use	
Smoking	14 (45%)
Multiple modes	13 (42%)
Vaping	2 (7%)
Edibles	1 (3%)
Dabbing	1 (3%)
Where cannabis is purchased	
Dispensary	22 (72%)
Online	3 (10%)
Friend	2 (6%)
Dealer	2 (6%)
More than one source	2 (6%)
Received mental health services	
Yes	26 (84%)
No	5 (16%)
Received services that are addiction-focused	
Yes	27 (87%)
No	4 (13%)
Received services that are cannabis-specific	
Yes	5 (16%)
No	26 (84%)
Level of involvement of parents/caregivers in services	
Not at all involved	18 (59%)
Slightly involved	9 (29%)
Moderately involved	2 (6%)
Very involved	2 (6%)

**Table 3:** Parent perspectives on mental health and substance use (n = 26)

Mental health and substance use of parents & their youth	n (%)
Parent has concerns about their own mental health	
Yes	11 (42%)
No	13 (50%)
Prefer not to say	2 (8%)
Parent has concerns about youth’s mental health	
Yes	25 (96%)
No	1 (4%)
Youth has diagnosed mental health concern	
Yes	21 (80%)
No	3 (12%)
Unsure	1 (4%)
Prefer not to say	1 (4%)
Youth is using other substances (in addition to cannabis)	
Yes	13 (50%)
No (only cannabis)	9 (35%)
Unsure	4 (15%)
Parent has concerns about youth’s cannabis use	
Yes	24 (92%)
No	1 (4%)
Missing data	1 (4%)
Youth has problematic gambling	
No	17 (66%)
Yes	4 (15%)
Unsure	4 (15%)
Missing data	1 (4%)
Youth has problematic gaming or tech use	
No	16 (61%)
Yes	9 (35%)
Missing data	1 (4%)
Youth received mental health services	
Yes	23 (88%)
No	2 (8%)
Missing data	1 (4%)
Youth has received addiction services	
Yes	8 (31%)
No	15 (57%)
Unsure	2 (8%)
Missing data	1 (4%)
Youth has received services related to cannabis use	
Yes	12 (46%)
No	11 (42%)
Unsure	2 (8%)
Missing data	1 (4%)
Services or treatment for youth involved family	
Yes	9 (35%)
No	14 (53%)
Missing data	3 (12%)
Parental involvement in youth’s treatment or services	
Not at all involved	4 (15%)
Slightly involved	10 (38%)
Moderately involved	3 (12%)
Very involved	8 (31%)
Missing data	1 (4%)

### How do participants describe the perceived risks or harms associated with cannabis use?

**Fig. 1:**
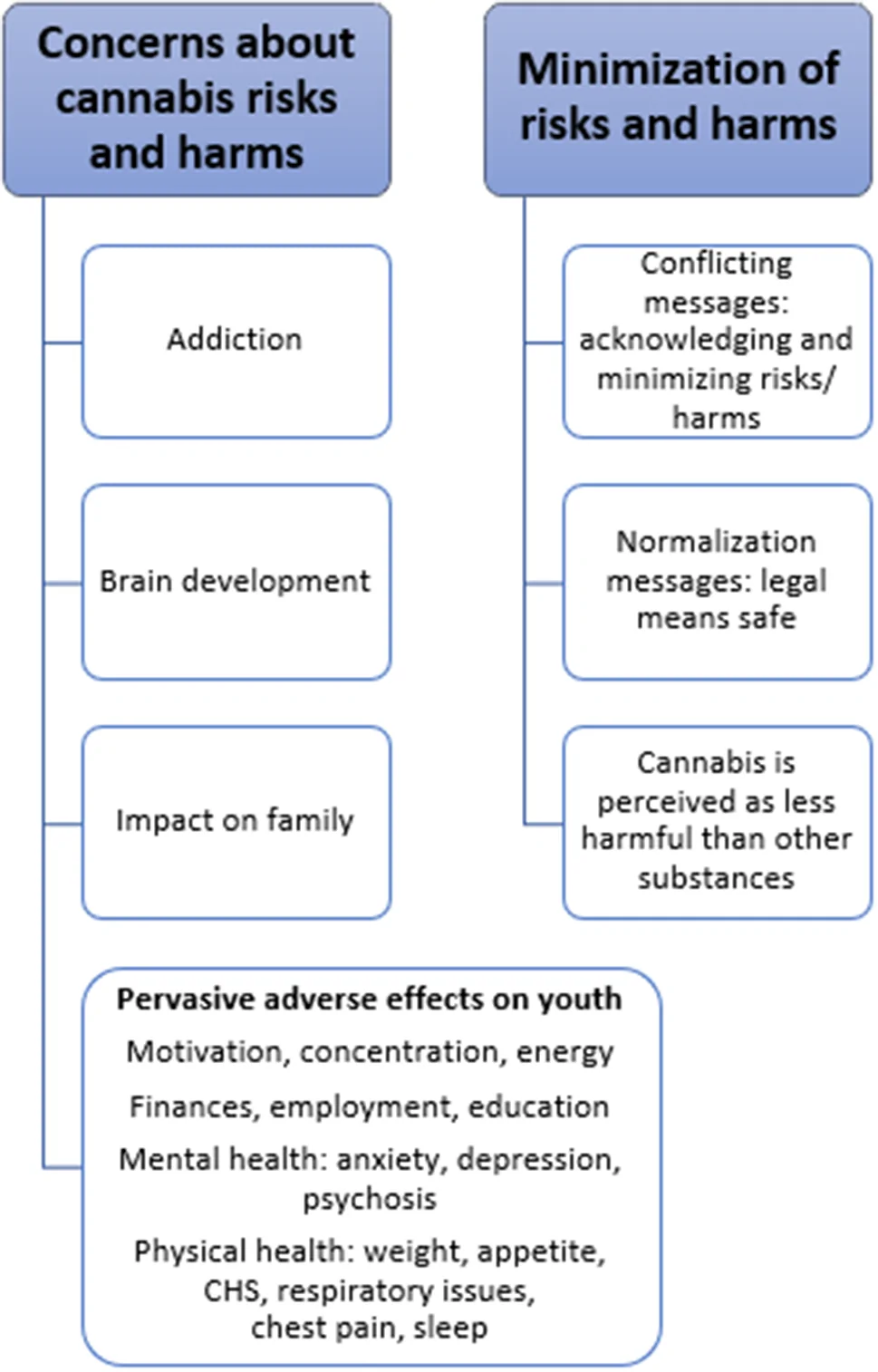
Themes and subthemes about perceived risks or harms of cannabis use

Responses from participants across the three groups were categorized into two main themes, each comprising more specific subthemes: (1) concerns about cannabis risks and harms; and (2) minimization of risks and harms. Figure 1 illustrates the overarching themes and their corresponding subthemes.

#### Concerns about cannabis risks and harms

Nearly all participants from the three subgroups expressed concerns about the risks and harms associated with cannabis use among youth, with the exception of two youth participants and one service provider. The emerging subthemes included concerns about cannabis addiction or dependency in youth, the impact of cannabis use on brain development, its effects on families, and its pervasive adverse effects on areas such as motivation, concentration, energy, finances, work, school as well as mental and physical health.

Participants underlined that there is an “underwhelming amount of conversation around the risk of dependency” (P17). Many youths described using cannabis to manage their mental health concerns, and not being able to determine if cannabis is helping considering the state of dependency on the substance: “I find that it helps with my anxiety, but of course now that I’m dependent on it, I’m not sure if it really helps with my anxiety or if I’m just in a state of anxiety with all my weed” (Y25). A parent described the severity of her son’s addiction to cannabis, expressing concern that others do not fully understand the extent of its potential harm and addictiveness:

This is probably the number one problem with our oldest son. We’ve almost lost him a couple of times due to cannabis addiction. I don’t think anyone understands how addictive it is, of a substance, and what can happen to the brain of a youth. It’s an absolute horror and a nightmare, what we’re going through. We can’t get him the right type of help, because I don’t think anybody really understands how serious this particular drug is. For our son, it is as severe and as dangerous as any other street drug and he has tried them all. This is what he keeps coming back to and it has a grip on him and it’s fundamentally changed the course of his life at this point. It’s brought out violence in him…rather than it having the effect that you would think…people think it makes them chill. (P14)

This youth explained the addictive nature of “poppers” which were defined as a mixture of cannabis and tobacco smoked through a bong:

It’s very addictive. I had been smoking poppers for like 4 or 5 years, and it was like the last couple years that I really started feeling those health effects and really wanted to quit. I would keep telling myself like literally almost every single day, like this is going to be my last popper and uh, it was never the last popper, and I would continue smoking and continue telling my friends like I think I’m having an attack, I think I’m gonna die. I really need to quit. (Y15)

Concerns about the impact of cannabis on brain development in youth were voiced by numerous participants. For example, a service provider underlined the need “to understand how that impacts the growing brain” (SP15). Another statement by a parent reflected some of the uncertainty related to the risks: “it’s a mind-altering drug and kids are still growing who knows what’ll happen” (P21).

Another harm, primarily identified by parents and two service providers, related to the impact of cannabis use on individual family members and the family unit as a whole: “He had anger management issues…it became really hard for all four of us, for my wife and I, for my other son,…all the crap that was going on really made it stressful and not very enjoyable.” (P3).

A service provider described the challenging realities faced by families:

Cannabis misuse definitely has a dramatic impact on families, the number of families that have called us where the youth is living in the basement, not going to school, not working, and they’re walking on eggshells…I see a large number of youth who are violent when intoxicated and are violent when they’re withdrawing. (SP30)

Pervasive adverse effects on youth were highlighted by most participants, with significant impacts on motivation, concentration, and energy being among the concerns. Youth 2 stated, “It definitely affects my motivational levels when using, as after using cannabis, I feel quite lazy. I feel like it’s kind of hard to focus on one task.” Another youth stated, “I didn’t realize it at first but after a while, I noticed that this is actually taking a toll on me…It’s just this feeling of like kind of brain fog” (Y12).

The impact of cannabis on finances was raised by many youths and parents, as well as some service providers. They emphasized the inordinate amount of money spent on cannabis at the expense of not eating or paying for other necessities. One youth stated, “I don’t even wanna guess how much money I’ve spent…I’m gonna say it’s probably somewhere $10, 000, $20, 000 over the course 5–6 years” (Y25). Parents had similar comments. For example, this parent noted, “I’ve seen that it has impacted his savings, that he was motivated to save money for the future in the past, and so, now his disposable income goes towards the purchase of marijuana” (P1).

Although most youth noted effects on motivation, focus, and energy, most did not believe that their cannabis use affected work and school performance. However, many parents and some service providers stated that it had negative impacts and were concerned about youth using cannabis at work or school. A parent stated, “He did an apprenticeship in plumbing. He was on a high-rise building, the first week of work, and he smoked on the job, within less than two months, he was let go, he was fired” (P11). A service provider explained that the youth they serve often have academic issues: “the youth that I see in my work…these are kids who are either failing courses or dropping out of school altogether, they start increasing the amount of cannabis they use, they’re usually intoxicated for most of the day” (SP 25). A parent also explained the drastic change in her son’s academic performance after using cannabis:

When he started using it, he just - he dropped like a stone. So, he has ADHD and he’s gifted, and he went from being an A student to failing, like that, in a heartbeat. It went from, you know, sampling to extreme use, extreme use. Like, three, four times a day. And that’s all he lived for. And he just checked out. Checked out of school, checked out of everything. (P2).

Mental health emerged as another area of concern, with participants specifically pointing to anxiety, depression, and psychosis as adverse effects linked to cannabis use. According to this service provider: “temporarily it might be helpful, but long term it makes mood and anxiety disorders much worse, psychosis 100% worse. The earlier the cannabis use, the worse outcomes. 16–24 is so formative and can impact the rest of their life” (SP19). A youth described the effects of cannabis as follows: “I’m like torn between it makes me more depressed and anxious and I’m smoking because I feel depressed and anxious. When I’m high I feel fantastic but then when I’m not I feel like it makes me feel worse” (P13). Another youth stated, “my mental health was just awful, um like depressed and anxiety and induced like a panic attack a lot of the time… becoming dependent on it has induced more anxiety” (Y15).

Participants across all three groups highlighted risks and harms associated with cannabis-induced psychosis or psychotic disorders exacerbated by cannabis use. Seven parents (27%) reported that their youth had experienced at least one episode of cannabis-induced psychosis. One parent shared his family’s challenging experiences over the last few years: “Daily cannabis use led us through three-and-a-half, four years of hell, and it got to the worst point was when it created a psychotic break for him…I think he scared himself…that the psychosis had taken hold of him” (P3). Another parent noted, “he started having his psychotic episodes at age 16. Every time he goes through his psychotic episodes, he is smoking marijuana prior to that happening” (P7). Six youths (19%) expressed concerns about the risks of psychosis and two reported previous experiences with cannabis-induced psychosis. One shared, “If I had known that I had bipolar, and like weed can trigger uh, psychosis in certain people, I probably would’ve never tried smoking weed, ever” (Y10). Another youth stated, “I was hallucinating really scary things” while experiencing a cannabis-induced psychotic episode, which resulted in self-harming behavior with serious consequences (Y14). Furthermore, some participants shared that many youths continue using cannabis even after experiencing a psychotic episode. As one parent explained, “he started to develop psychosis at the time, and he would smoke more like to calm himself. He believed that he was helping himself just with cannabis and the more he smoked, the worse he was getting” (P17). A parent highlighted needing to call emergency services when her son has used cannabis and has symptoms of psychosis: “there’s a lot of times that we’ve had to have the police here and ambulance here” (P6). Service providers in emergency settings noted a significant increase in cannabis-induced psychosis since its legalization. One emergency service provider stated, “I don’t think it’s a commonly known thing that at the very worst like you become psychotic from using cannabis. That’s kind of minimized…all those things should be general education for any provider working with youth, it’s not benign.” (SP31).

Another emergency service provider highlighted the increased risk of psychosis among youth using cannabis with high THC levels:

There has never been the THC concentrations that we are seeing now…concentrations upwards of 15, 20, 30%…And when you have something like that the brain will react to THC very differently than it reacted to someone taking cannabis at a concentration below 3%…The higher the dose is consumed the relative certainty, that you’re going to push anyone into psychosis. (SP14)

Adverse effects on physical health emerged as another significant concern related to cannabis use among youth. Chest pain and respiratory difficulties were frequently reported by youth participants, a concern that was also noted by numerous service providers and parents. One youth highlighted, “I have noticed my lungs specifically being in a little bit of pain when I’m taking deep breaths and like I’m feeling that little bit of that lung congestion” (Y17). Another youth added, “when I cough it’s like, there’s like black stuff in my spit and then I just, I know I need to give my lungs a break. I’ll feel like I have to puke.” (Y21). Several participants from the three groups discussed severe bouts of vomiting known as cannabinoid hyperemesis syndrome (CHS). A parent reported that his daughter had multiple episodes of CHS, stating, “It’s just the increase of people going to the hospital like I didn’t even know this thing, Cannabinoid hyperemesis syndrome, but she’s put herself in the hospital probably four times in the last year” (P24).

Weight loss and changes in appetite were additional harms described by some participants, including this youth: “I was really weak, um tired all the time, uh and you know my appetite was just like not there. Weight loss was like huge, now I weigh about 200 lbs, like 200 to 210. At the time when I was smoking poppers, I weighed 160 and I’m 6’5…so 160 was really really skinny” (Y15). Similarly, a parent expressed concerns about weight and appetite in her son: “when you have chronic pot use, it also affects your digestive system, he’s not eating enough. He always has an upset stomach so he’s very thin” (P4).

#### Minimization of risks and harms

Responses from research participants revealed a paradox, with all three groups expressing substantive concerns about the risks and harms associated with cannabis use, while also minimizing its potential adverse effects. Minimization was most prevalent among youth participants (98%), followed by service providers (39%) and parents (8%). The subthemes emerging under the theme of minimization of risks and harms included conflicting messages, where risks and harms are both acknowledged and minimized; normalization messages, suggesting that legal means safe and harm free; and perceptions of cannabis as less harmful than other substances.

This quote from a youth underscores the conflicting perspective by acknowledging the symptoms associated with cannabis use while also expressing the belief that these symptoms do not pose a problem:

I get like chest pains and lung pains and just pains everywhere…if I smoke a little too much, my heart rate can go to like 140, 150…So, it’s kind of like something that *I don’t think is an issue*…[emphasis added] that’s how it’s impacted my physical health and when I smoke, I cough a lot like no matter which method of smoking I’m using…and that also hurts me. (Y19)

Similarly, another youth described their perceptions of the impact of cannabis on their physical health:

*I don’t think it affects my physical health all too much* [emphasis added]. It did affect my throat at first when I started smoking…my throat would be kinda raw. Um, and like my lung capacity…I used to run a whole bunch and now I can’t because like the vaping and smoking just like kind of like destroyed my lung capacity. But other than that, *I don’t think it has much of an effect on my physical health* [emphasis added]. (Y29)

Many service providers reported working with youth negatively impacted by cannabis, emphasizing that these youth often fail to connect their issues directly to its use. For example:

I do see a lot of people with cannabis use with that like persistent cough that they have you know where it keeps going. Also, I’ve seen over the years tragic cases you know where um drug-induced psychosis with cannabis has happened…I think *there’s a real resistance that cannabis is causing these issues* [emphasis added]. (SP2)

One youth admitted to having an addiction but also stated that it is not harmful:

I do feel like I’m addicted to it, and I don’t wanna say that like any addiction is really like a good addiction, but *I feel like it’s not necessarily a harmful one* [emphasis added] where it doesn’t really affect like my, my days really. I mean I guess it sort of does cause I’m waiting to go home and use it, but in a way like it doesn’t affect my work and it doesn’t really affect many of my relationships in a way that stresses me out a lot. But I would say that I’m addicted in a way where I can’t really sleep if I don’t have it. (Y16)

Some service providers did not have concerns about the negative effects of cannabis use in youth. For instance, one service provider stated, “*I can’t say I’m actually overly concerned about health* [emphasis added]. I think there’s some concern to it, but *there’s other things that concern me more* [emphasis added] in terms of health” (SP 6). While the majority of parents expressed serious concerns about the potential risks and harms from cannabis use, a few parents held conflicting views. One parent said, “*I don’t think that it is affecting his physical health* [emphasis added], right at this moment,” yet also shared, “I would prefer he wasn’t inhaling smoke all the time. I believe that will catch up with him… he had a panic attack after consuming cannabis” (P 23).

Several participants attributed the minimization and denial of risks to the normalization of cannabis use which amplified after legalization, and the widespread message that legal means safe and harm free. Illustrating this point, a youth stated,

I think it’s just become so normalized, that it’s a thing that people should be doing. And especially with like the legalization of it, and like you know pot shops like popping up everywhere like I think we have more dispensaries than we do like coffee shops in [Northern Ontario]. It’s just so prevalent that I don’t think um, it crosses people’s mind that it’s something that could be like bad for you, and it really kinda gets this rap that it’s really beneficial, a good coping mechanism to like relax and stuff. (Y30)

A parent reflected on how his son’s use of cannabis has been normalized and its harms minimized:

He considers that it has medical properties, and those medical properties are the same as taking a prescription medication. It seems to have been normalized for quite some time. Normalized and any harm is minimized. You know, this idea that it’s a natural substance and you know, that’s not going to bring you harm, and a narrative about it being medical. (P11)

A service provider supporting young parents of infants normalized parents’ use of cannabis while minimizing the risks to young children:

They don’t have a lot of support and by using marijuana it helps them feel like it’s something that just takes the edge off. They’re not necessarily trying to get high or not trying to be in a capacity that they can’t care for their kids. As long as you can use it safely, and it’s not impacting your parenting, it’s not impacting the child. (SP12)

Many participants argued that the abundance of misinformation and normalization, coupled with the lack of public education, contributes to the perception that legalization of cannabis means harm free. For example, this service provider explained,

I mean it’s very easy to get them off cocaine or get them off anything serious. It’s not easy, but the education is easy. But weed…because of this legality, it’s becoming socially acceptable. They’re just thinking you know what’s the big deal? I mean I’m just smoking weed you know. But that weed brought you to the hospital three times! Like you’re not putting the equation together? (SP4)

Another subtheme that emerged involved comparing cannabis to other substances, with cannabis often perceived as less harmful. This perspective was supported by a service provider who suggested that for youths with a history of trauma, the use of cannabis is not the primary concern:

My reaction to that is going to be much different than for a 16-year-old kid who has an extensive, extensive trauma history, and her using a little bit of cannabis on the weekend in terms of harm reduction is the least of the things that she could be doing to manage her sleep or her anxiety…I don’t really have a problem with it. (SP9)

One youth described cannabis not having the same effects as cocaine:

I had tried just a tiny little bit of cocaine…my heart was racing, and I wasn’t a big fan of that like feeling of not being able to relax or be in control of like how I was feeling. which is why I think on the other side of things, I kind of like weed because like you can enjoy like doing other things…you also could just go right to bed and have a really good deep sleep. (Y13)

Another youth spoke about feeling that cannabis use is less stigmatized than other substances, even for youth who are under the minimum legal age:

I’m more comfortable because I feel it’s less like problematic. Like it hasn’t cost me as many issues as the other substances have. And also because it’s legal, even though I’m not of age, um I’m still doing something that’s legal…Individuals who have certain views on people who use drugs and stuff. They don’t have those views on people who are smoking weed. (Y19)

A service provider expressed a view similar to that of the youth, emphasizing the different way cannabis is perceived compared to other substances:

With youth especially there is the hierarchy of substances. I think it’s really hard for youth to get support with solely cannabis use because I think often in recovery communities, there’s a bit of like annexing…with weed, is it actually an addiction? Youth continue to feel even like stigmatized or undermined, if they do get to the point of saying, okay, I am wanting to make some changes and then feeling a bit kind of pushed out of those recovery communities. (SP13)

### How do participants describe their own and others’ approaches to reducing cannabis-related risks and harms?

**Fig. 2:**
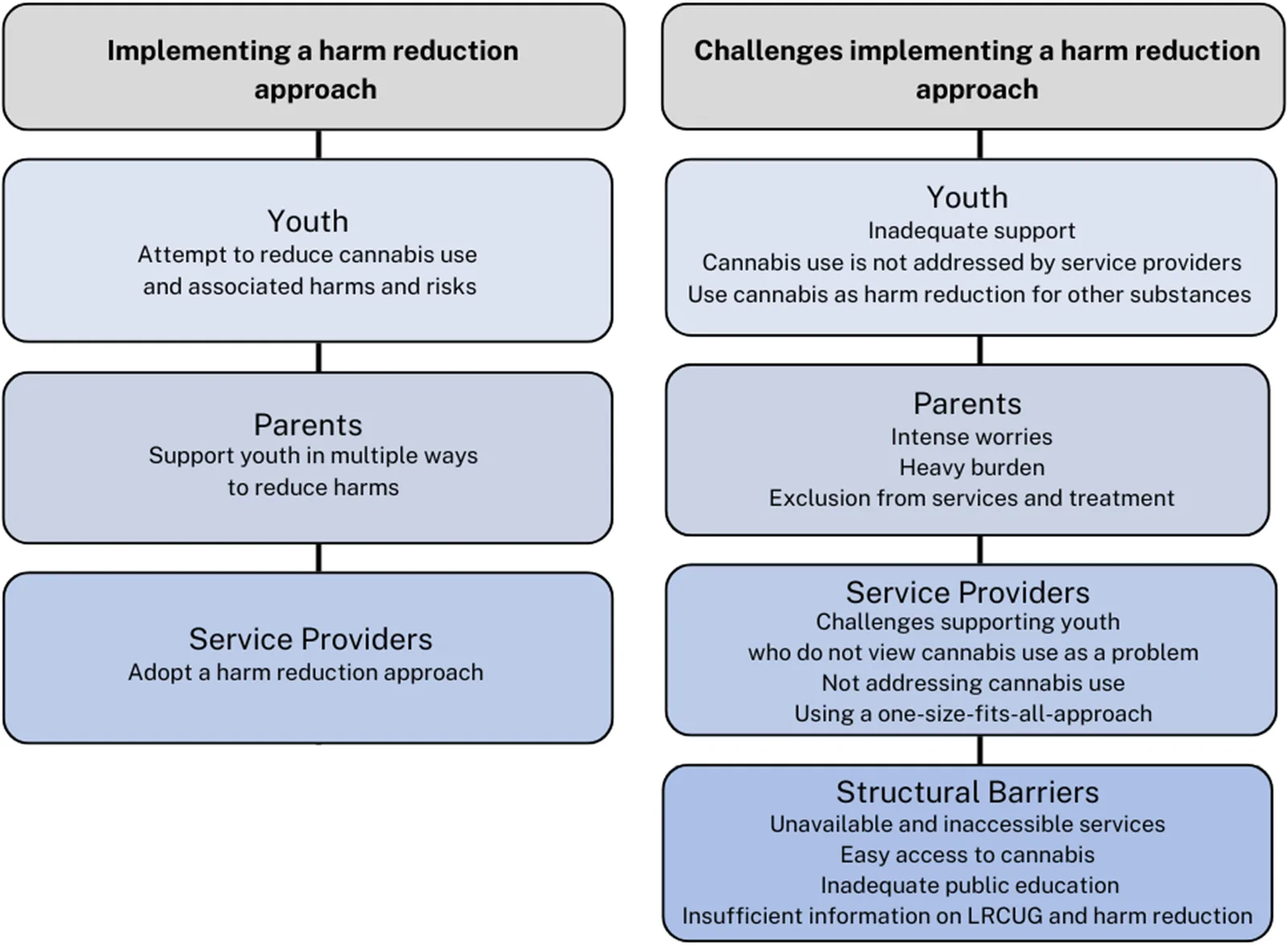
Themes and subthemes about approaches to reduce cannabis-related harms

Two main themes emerged from how participants described both their own and others’ approaches to reducing cannabis-related harms: (1) Implementing a harm reduction approach, and (2) Challenges in implementing a harm reduction approach. Each theme includes subthemes that detail specific strategies and challenges encountered. Figure 2 shows the themes and their corresponding subthemes.

### Implementing a harm reduction approach for cannabis

Under the theme “Implementing a Harm Reduction Approach,” three subthemes emerged, describing specific realities for each participant subgroup: (1) Youth: most have tried to reduce the harms of cannabis use; (2) Parents: all support youth in multiple ways to reduce harms; and (3) Service providers: most adopt a harm reduction approach.

#### Implementation for youth

Among the participants, 12 youths (39%) discussed various measures they have taken to reduce cannabis use or mitigate its associated harms. Most of the youth who attempted to reduce or stop their cannabis use found they were more negatively impacted with some describing feelings of dependency. Some had specific strategies to reduce their use, such as changes in frequency, cannabis type, or time of the day. For example, this youth emphasized,

It’s dropped drastically, I used to smoke when I woke up, and then smoke during the day and then smoked at night…I found it really diminished some of my motivation, so I’ve cut back quite a bit and I’ve now gotten to the point where I just use it at, once maybe twice a day. And I don’t smoke in the morning anymore. It’s usually just an at night thing. (Y31)

Two youths reported that they stopped smoking and are trying to use only edibles (Y28 & Y29). Youth 29 explained needing to learn about how he was affected by different products: “When I was younger…I was going with whatever I could get. Whereas now I’m learning about how different strains affect me…Now I’ve just fully stopped smoking cannabis.” (Y29).

Another youth described several limits set to reduce harms:

During the school year I would always only smoke after 8 pm so that I had all the time after class to do homework…For work obviously I never go to work high cause I can’t drive and um, it does make me sleep in when I do it before work. (Y18)

Another youth mentioned that “I started moving towards that because I, I don’t like smoking it as much. I’ve noticed it’s a lot easier to control the edibles versus the smoking cause I find that smoking affects me like differently” (Y30). Three youth mentioned taking “tolerance breaks” (Y2, Y14, Y16), suggesting a period of non-use.

#### Implementation for parents

All parent participants described using diverse strategies to support their youth and help them mitigate the harms of cannabis use. Eight parents (31%) also spoke about harm reduction strategies used by their youth. Parents detailed various approaches to support their youth, including facilitating connections to services or treatment, advocating for access to appropriate and timely services, and providing emotional, financial, social, and instrumental support. They also emphasized the need to respond to crises, support other children impacted by their sibling’s cannabis use, educate themselves about mental health and addictions, and provide their youth with information. One parent shared,

It’s very hard as you go through it, to try and find services where we can educate ourselves also. So, it’s just having resources that are available for the caregivers, because when the person is going through the psychotic episodes, they’re obviously not in a position where they can think clearly and make decisions. It’s the caregivers that need the resources, you know, that need to be given the information and they need to be educated so they’re in the right position, they have the right information to be able to then help or get the help that the individual needs. (P7)

Another parent expressed frustration over service providers downplaying the risks of cannabis use and not taking parents’ concerns seriously:

There’s a real resistance to a mother saying my child is not well, and I need help. There’s a real tendency to think, she just needs to see a therapist and talk it through. We had to fight with her GP for a referral to addictions, we were told she was just a normal teenager…we really did not receive services until we fought and fought and fought for them. (P13)

Some parents took steps to obtain a form for the involuntary admission of their youth to a hospital, seeing it as a necessary measure to reduce harm. One parent shared concern about her youth posing a risk of harm to himself or others due to violent behavior linked to cannabis use:

He wants marijuana over everything else. He’s been formed twice and placed in the mental health units at the hospital. And when I say violent, he gets extremely violent, he’s a danger to himself and he’s a danger to the community. (P14)

#### Implementation for service providers

Most service providers (94%) reported adopting a harm reduction approach to cannabis use. However, only 12 (39%) explained how they implement this approach. The most common harm reduction strategy service providers described was psychoeducation. This emergency department service provider stated, “I provide some education with a harm reduction approach…that conversation with patients when they are in the emergency department about cannabis withdrawal and how there can be heightened states of anxiety several hours after the consumption of cannabis” (SP14). Another service provider added, “I use a harm reduction approach, and provide education about you know harm versus not harming and ask people to buy cannabis from a legal place as opposed to somebody that is selling it” (SP1). A third service provider stated, “there’s a part that is psychoeducation…how the cannabis use can be impacting their mental health and letting them talk about what are their beliefs around cannabis, what do they think is helpful for them with cannabis. And start talking about alternatives…maybe not stopping using…just delaying use” (SP25). One service provider noted that their own lived experience was a helpful medium to reach youth about the harms associated with cannabis use: “Having my own struggles with it like adds to the fact that I can speak to it a bit more like authentically and it’s not going to come across as preachy” (SP17). One service provider spoke about proposing alternative coping strategies to youth: “Reduce the use or just like encourage different ways and different means of coping…If they’re smoking every single day because they’re stressed and they’re unable to calm their minds which we get a lot…offer other suggestions” (SP21).

### Challenges in implementing a harm reduction approach

The theme “Challenges in implementing a harm reduction approach” encompasses four subthemes, each describing specific challenges for each participant group, as well as one subtheme related to structural barriers that cut across all three groups. The challenges are as follows: (1) Youth challenges include inadequate support, experiences where cannabis use is overlooked or dismissed by service providers, and using cannabis as harm reduction for other substances; (2) Parent challenges include intense worries, heavy burden, and exclusion from their youth’s treatment; (3) Service provider challenges involve difficulty supporting youth who do not view it as a problem, not addressing youth cannabis use, and using a one-size-fits-all-approach; and (4) Structural barriers include unavailable and inaccessible services for youth, easy access to cannabis, inadequate education on cannabis risks and harms, and insufficient information on LRCUG and harm reduction.

#### Challenges for youth

The first subtheme outlines challenges experienced by youth associated with reducing cannabis use. Many youths described not having support and attempting to reduce their cannabis use alone. One youth explained that they had experienced many unsuccessful attempts to take breaks from cannabis use: “I am somebody who continues to smoke weed but like desire to stop smoking weed…I think it just proves how addictive it is… I just wish that I could control and moderate it, for my own benefit” (Y15). Another youth underlined that they had been using for a long time making it even more difficult to stop: “I’ve been smoking since grade 7 and it’s been pretty hard to quit. I tried many times but to no avail. I’ve been using it for such a long time…Never try it…I wish I’d personally never tried it that day.” (Y6).

Some youth highlighted that their concerns about cannabis use were overlooked or dismissed by service providers. For example, this youth felt unsure about raising cannabis use with their therapist, due to its minimization and stigmatization:

I had brought it up in therapy once to my therapist…She hadn’t brought it up until I had mentioned it… Um, I feel I felt embarrassed at first to be bringing it up because it just seemed silly because you know in therapy these are not the kinds of things that you should be talking about. I was telling her how I’d been smoking a lot and you know I feel like it’s maybe, it’s too much…She said, ‘well if you’re aware of the reason why like you know for the time being you know if it helps you sleep you can continue that’…Then we never really revisited it cause I never brought it up again…finding a professional who’s a little bit more in tuned in…can actually help you to make the changes you’re looking for. (Y13)

Another youth shared their experience with emergency services, highlighting a perceived gap in care due to a lack of familiarity with cannabis-related issues:

I feel like there should be at least in my opinion, somebody that works in emerg that’s well-versed in substance abuse and if I want to quit or I want to cut down, I can go to the emergency room and access somebody. I feel like though when it comes to cannabis, they don’t have people that are knowledgeable about that. (Y22)

For some youth, reducing cannabis-related harms was further complicated by their use of cannabis as a self-prescribed treatment or a means to reduce their use of other substances as explained by this service provider: “I hear from a lot of youth it’s the substance to use if you’re trying to stop other substances, they’ll say it’s the least harmful if they’re using other substances and then, the one that sticks around forever” (SP13). A similar statement was made by another service provider: “They’ll cut everything off except for cannabis. Youth are using it to make do with their mental health to really almost treat that…they use it as like a harm reduction to when they can’t afford other substances” (SP15). One parent described how her son stopped using opioids but continued using cannabis and alcohol: “[Son] overdosed again. just over a year…So, now he just smokes weed. And he’ll drink and smoke weed pretty much every day” (P2).

One youth described being abstinent from an addiction to prescription medication and explained that despite a cannabis-induced psychotic episode that had serious and negative outcomes, they have had challenges reducing their cannabis use: “I’m California sober. It’s essentially being sober, but you can smoke weed” (Y14). This youth added, “I really had to fight to get proper services” indicating that cannabis is not a subject that is discussed with most service providers.

#### Challenges for parents

The second subtheme focused on the challenges parents face in supporting a youth to reduce the harms associated with cannabis use. These challenges include intense worries about the risks to their youth’s health and safety, a heavy burden, and exclusion from services and treatment. This parent described the intense worries and sense of helplessness: “I can’t change [son]. I can try to do the things that I’m doing to support him…help him come to a place of better decisions…I don’t actually want to know - the rate at which he’s degrading his cognitive ability and that it’s irreparable.” (P11) Another parent shared the impact that her daughter’s cannabis use was having on her: “It’s been an unchartered territory for me. Something that I see as a monster, as an ugly thing. Something that I didn’t want it for my daughter. I think I cried for 5 days in a row” (P19).

Participants described inadequate professional support for parents and exclusion from their youth’s services and treatment as noted by this parent:

I think they could talk to the [youth], and say, we all want to help you and it would be really helpful if we could have at least one session with your parents. Maybe they view the parent as somebody who is making things worse. There’s a lot of blame for parents when it comes to any kind of mental health or addiction and that’s not helpful in any regard. (P10)

A service provider explained the reluctance to involve families:

Sometimes we over rely on families to participate in the change and sometimes we under rely on families to participate in the change. Sometimes I think service providers bring families in as leverage. Like, I can’t get the kid moving…you get the kid moving. If your experience with family members is that they’re really overbearing and they you know pushing for change when the kid’s not ready, you’re more reluctant to involve them because you see them as a negative contributor to a child being able to make change. (SP28)

Some parents stated that there are cultural differences in Canada compared to non-Western countries that involve parents in healthcare: “I do think that this young age for kids to make their own decisions is not right. In our country, parents continue to talk to the doctors” (P19).

#### Challenges for service providers

The third subtheme focused on challenges for service providers including how to help youth who do not perceive their cannabis use as problematic. This service provider explained, “Their parents are sending them to see me because of their cannabis use and they’re willing to talk about anything but their cannabis use…It’s seen very much as a solution and not much interest in making changes around it” (SP2).

Many service providers reported not screening, assessing, or offering other services related to cannabis use among youth, while also stating that they adhere to harm reduction approaches. For example, two service providers stated, “I don’t ask about it unless they bring it up” (SP10, SP22) with one adding, “I only ask about it if it’s related to the actual presenting issue” (SP22). Another service provider highlighted, “We don’t go too much into that because we’re not like medical doctors, so we don’t want to be too intrusive and make our clients feel uncomfortable” (SP5).

Some parents and youths raised concerns about the use of a one-size-fits-all-approach by some service providers, which does not address the needs of all youth receiving services. Parents noted that many services providers do not consider abstinence as part of harm reduction, and only two participants from the service provider group mentioned that abstinence is included in a harm reduction approach. A parent underlined, “What a lot of people don’t understand about harm reduction is it’s a continuum and they always seem to negate the abstinence factor as an option” (P4). Another parent explained that the approach used by the therapist was not suitable for her youth: “The private therapist that we’re paying for…very much a harm reduction approach, which I get, from a professional standpoint, but it does not work for this child” (P16). Similarly, another parent questioned the effectiveness of approach used by the treatment center for her daughter:

[Addiction treatment center] is big on harm reduction. So, they don’t encourage teenagers to stop using completely. Maybe that works for the vast majority of who they’re treating, but it did not work for our daughter. Our daughter had a serious addiction, and they would not back us…It was counter to our child’s recovery. (P13)

### Structural barriers

Participants identified several structural barriers that pose challenges to reducing risks and harms of cannabis use among youth. These include unavailable and inaccessible services for youth, easy access to cannabis for youth, inadequate public education on the risks and harms linked to cannabis use, and insufficient information on lower-risk cannabis use guidelines and harm reduction.

Many participants described the lack of appropriate and on-demand services as an additional challenge to reducing the risks and harms of youth cannabis use. Some participants from rural communities including Northern Ontario explained that there was an even more pronounced lack of accessible services in their communities. One parent highlighted the immense pressure parents face when they are concerned for their youth’s safety and no available services are available:

Least helpful is how few options there are for a kid like him in a time of need. The hospital couldn’t control him…It felt life-ending at different points because we were just sitting there saying, like, now what? Nobody wants to help him. Nobody thinks they can manage him. What do we do now? The waitlists, just hideous. When a kid’s in need, it needs to be fast. We just tried to keep him safe for 30 days. It was just torture because we were waiting for a spot. We’re paying for all of this. And it was just to try to keep our child safe…He’s been in jail a couple of times, that’s not where I want him, but at least I know he’s safe. (P14)

A youth also explained the need for cannabis specific residential services:

I personally would benefit from, if there was like a site that I could stay at that I would be able to like get off of smoking weed and vaping. I feel like it needs to be a program that’s specifically for you know smoking weed and/or vaping…you know like somewhere comfortable that doesn’t look like a hospital. They would teach like you know coping mechanisms and sort of look at the issues of why people smoke weed. (Y19)

A parent also underlined the need for cannabis specific intensive treatment: “All I can think is that it must need specialized treatment. It just doesn’t seem to be something that he can detox off of. And he’s tried” (P14).

Many participants described the challenge of implementing harm reduction when cannabis is readily available and easily accessible to youth. They argued that the minimum legal age is too low, that there are too many cannabis retail stores, and that it is too easy for youth to purchase cannabis in stores and online even if they are below the legal age. This youth underlined, “how accessible it is needs to change…I have a baby face, if I can walk into a dispensary and buy at 16, there’s a problem. It shouldn’t be that easy for me to get it” (Y24). Another youth stated, “cannabis should be legal for people that are 25 and over. For me having started smoking at a young age…it should be like restricted to a higher age, so that you don’t run the risk of having bad development” (Y28). A parent reported, “the cannabis stores are everywhere! And they break all the rules” (P12).

Most participants identified the lack of public education on the risks and harms of cannabis use as a significant challenge in harm reduction, as highlighted by this parent: “I would like to see more information provided to young adults about harm reduction and ways to reduce their harm” (P1). A youth echoed this message emphasizing the need for an “empowered toolkit of harm reduction strategies” that could help understand “safety tips” and answer questions such as “why is it bad? Why does it hurt your lungs? Why might it affect your prefrontal cortex? Why might it lead you to smoke every day?” (Y17). Another parent added, “it’d be just really important to be able to kind of educate the youth about that and uh, at least give them an understanding of it before they decide to experiment with it, if at all” (P8). This service provider added:

I think there’s a lot of misinformation…the cannabis industry is using kind of the same strategies that the tobacco industry used. There are a number of websites where they provide all these benefits that are not necessarily substantiated in any way. There wasn’t a really thought-out process when it was fully legalized. This is disconnected because in the political discourse having an addiction is about individual choice when actually addiction is not a choice. The government has been walking a very fine line in terms of substances. It took so long to recognize, for instance, the harms from tobacco. We know the harms from alcohol, and yet we have governments who encourage alcohol consumption. For cannabis, there needs to be education and recognition of potential harms. (P25)

Most participants underlined needing more information on LRCUG and only one of the participants from the three groups reported having knowledge of LRCUG for youth or adults. A parent stated, “there really need to be guidelines on what is reasonable and decent and safe use for cannabis…None of that seems to exist for weed. Maybe it does? But it’s certainly not apparent” (P12). A service provider added, “What is considered safe and what’s not? We have safe drinking guidelines for alcohol, with cigarettes, no amount of smoking is safe. With cannabis, I’m not sure that we have that and if there is it’s not well disseminated” (SP29). A few service providers noted that clearer safety and harm reduction guidelines would influence service provider knowledge and consequently, the level of care. For example, this service provider underlined:

I don’t actually really have any concrete like guidelines to go by and I do have um, like teenaged clients that are kind of going through this…this is such a big problem…and I was like wow, I actually don’t like have concrete kind of guidelines to go off of on like when is it a problem versus when is it not. (SP5)

A parent also expressed wanting to understand how to reduce the harms: “the whole idea of harm reduction, you know, what does that look like? And what would be something safe versus unsafe?” (P10). Insufficient education on harm reduction was also underlined by this youth: “Teach people how to use it properly…Some people sit there, and they’ll smoke from a pipe that’s black as all hell… they over smoked their bong so much to the point there’s tar literally coming out of their esophagus” (Y9).

## Discussion

This study explored how participants describe the perceived risks and harms associated with youth cannabis use, as well as their own and others’ approaches to reducing cannabis-related risks and harms. Participants shared insights about various risks and harms, including dependency or addiction, concerns about brain development, negative impacts on family, and adverse effects in areas such as motivation, concentration, energy, finances, employment, school, and physical and mental health. While most acknowledged some level of adverse effects, there was also a tendency to minimize and normalize these effects. Our analysis of the challenges expressed by participants underscores the need for increased support and enhanced efforts to reduce structural barriers. These barriers pose challenges for participants in reducing the harms of cannabis use, whether for themselves or in supporting others. The identified structural barriers include unavailable services, easy access to cannabis, inadequate public education on the risks and harms of cannabis use in youth, and a need for more information on LRCUG and harm reduction strategies.

Our findings highlight several important areas that need attention to effectively reduce the harms of cannabis use among youth in Canada. First, there is an urgent need for enhanced public education on the risks of cannabis use and the LRCUG. This need for more information and education was described by all participants and aligns with the results of the CCS, which found that half of Canadians have not seen any educational campaigns or public health messages about cannabis [2]. Additionally, the CCS reports that among the topics related to cannabis, Canadians feel most uninformed about the health and safety risks with youth aged 16 − 19 and young adults aged 20 − 24 expressing the greatest desire for information on these safety risks, more so than adults over 25 [2].

The LRCUG were recently updated to provide more comprehensive information about health risks and safety. While these are not youth-specific guidelines, some recommendations specifically address risks for youth. Researchers emphasize that these guidelines can influence perceptions of cannabis use post-legalization and assist youth in making safer choices. They advocate for knowledge translation strategies to facilitate the dissemination and implementation of the LRCUG, which can be integrated with other prevention and intervention programs [36]. Most recently, a group of international experts formulated the lower-risk cannabis use guidelines for psychosis (LRCUG-PSYCH), which include 11 evidence-based, public health-oriented recommendations to mitigate psychosis-related risks associated with cannabis use [14].

Public education will also help address the normalization of cannabis use, which can be linked to reduced perceptions of risks and harms. Research shows that increased perceived risk and health knowledge reduce substance use and harms [58–60]. A review examining cannabis knowledge and risk perception, found that increased knowledge of cannabis was associated with an increased perception of risk and lower current use. Conversely, increased youth cannabis use was associated with lower cannabis-related knowledge and decreased perception of risk. The review recommends implementing public health strategies directed at youth with the aim of increasing knowledge, which could influence rates of cannabis use [58].

Studies have also found that normalization of cannabis and other substances can influence perceptions of risks and harms [61, 62]. While normalization can destigmatize substance use and encourage youth to discuss their use, it must be balanced with adequate harm reduction information to avoid promoting use [61]. The “normalization thesis” by Parker et al. [63] outlines how a drug becomes less stigmatized through shifts in cultural attitudes, policies, and accessibility. Abridge et al. [64] studied how tobacco moved towards denormalization, driven by comprehensive policy and educational changes, such as adjustments in marketing and smoke-free policies, which reshaped societal norms. The authors emphasized that public education and people’s perceptions of health risks are key factors in their experiences of normalization and denormalization [44]. 

The importance of public education was reported by the Task Force formed by the Government of Canada before legalization. To mitigate risks to youth under 18 and young adults up to age 25, the Task Force recommended that governments do all they can to “discourage and delay cannabis use” with “robust preventive measures, including advertising restrictions and public education” [4, p. 17]. The Task Force consulted with experts who strongly recommended a public health approach and an “evidence-informed public education campaign…with an emphasis on youth, parents, and vulnerable populations” [4, p. 26]. A review of public health approaches to substance use by Crépault et al. emphasized that for jurisdictions that have legalized cannabis, an effective public health approach should: “include the best available evidence, aim to reduce harm by focusing on and addressing modifiable risk factors, e.g. age of initiation and intensity of use, minimize the commercialization of cannabis, and include regulation, e.g. of price, availability, and marketing/promotion of cannabis” [68, p. 9]. The review pointed out that despite the Government of Canada’s claims of implementing a public health approach for cannabis, many key aspects of this approach have not been fully realized.

In 2022, the Government of Canada appointed an Expert Panel to conduct a legislative review following cannabis legalization. The panel expressed concerns regarding youth cannabis use and emphasized the need for strategies to reduce its prevalence among youth, develop more evidence-based programs, enhance regulatory measures to restrict access, and allocate funding for research. The Expert Panel concurred with the pre-legalization Task Force’s statement, which stressed that “it would be a mistake for governments to adopt an attitude of complacency with the current regime or move away from a public health and public safety approach to cannabis” [69, p. 2].

Another critical insight from our findings is the significant impact of cannabis use on parents and families, underscoring the importance of supporting families, and involving them in services and treatment. Research has shown that substance use disorders can create significant stress and strain in families [70, 71]. Our findings align with addiction literature that shows how families experience structural discrimination within systems that expect them to provide support for their children and loved ones but implement policies and services that do not adequately support family involvement in treatment and care [70–73]. Given the crucial role of parents and caregivers, it is vital to develop educational resources on cannabis use specifically for families. Involving families in the creation of these resources is also beneficial. Jenkins et al. [44] performed an environmental scan of existing cannabis education resources for parents and discovered that only 31% of these resources were Canadian, and while 16% involved youth in their creation, none included parents or caregivers.

In our study, a high proportion of youth participants were sexual and gender minority youth (SGMY), with 69% identifying as lesbian, gay, bisexual, or pansexual, and 29% identifying as gender diverse, trans, or nonbinary. SGMY experience higher rates of poor mental health and psychological distress, often attributed to discrimination, homophobia, and transphobia [65–67]. Recent studies have underscored the importance of contextualizing cannabis use within the social and structural conditions that perpetuate stigma and discrimination for SGMY and worsen mental health concerns linked with substance use. More research is needed to understand cannabis use among SGMY using an intersectional lens to better understand the complex interactions of gender, sexual orientation, race, and other social determinants that influence their experiences and health outcomes. A scoping review on youth cannabis use in Canada since legalization found that none of the studies examining cannabis use among youth collected data on sexual orientation, and most presented gender in a binary manner as either male or female [18].

One final result to highlight is the discrepancy among service providers in their knowledge of cannabis and how they address its use in practice. Most service providers in our study endorsed harm reduction approaches, yet many did not routinely screen or address cannabis use among the youth they serve. Several acknowledged the challenges of helping youth who do not perceive their cannabis use as problematic. Additionally, some youth participants reported that their cannabis use is overlooked by service providers, a finding also reported by Turuba et al. [74]. This aligns with the findings of another study exploring service providers’ perceptions and practices regarding youth cannabis use (*N* = 160). The study found that while most service providers believe that cannabis use poses potential harms and risks for youth, less than half discuss cannabis use with youth. Only 16% of service providers reported being familiar with treating cannabis use, and 67% seldom involve families in youth treatment [62]. This underscores the need for enhanced education and training for service providers.

### Strengths and limitations

The study has a few limitations that must be considered. The findings may not be fully representative of other populations outside of the context of this study. While our youth group was diverse, our samples of parents and service providers lacked diversity in terms of gender, race, and geographical location. The data presented is based on self-reports from participants, which could introduce biases such as social desirability. We also recognize that the researchers’ biases might limit the interpretation of the data. To minimize bias, we employed strategies such as using multiple data sources and investigator triangulation. Additionally, we held weekly debriefing meetings as a team to discuss our interviews and interpretations of the data, and we wrote memos after interviews to encourage reflexivity on how our own perceptions may influence the research process.

The study also had several strengths including its sample size and being one of the first Canadian studies to examine cannabis use from the perspectives of three key participant groups. Although we focused on Ontario, we had representation from across the province even though there were higher concentrations in Central Ontario. Our youth group was diverse in terms of race, religion, ethnicity, gender, sexual orientation, age and living arrangements , allowing us to hear from youth with a range of experiences.

## Conclusions

This study highlights perceptions of the risks and harms associated with youth cannabis use from the perspectives of youth, parents, and service providers. The findings have significant implications for policy, services, education, and training. Youth cannabis use is a major public health concern that must be addressed through a multi-pronged public health approach. This approach should prioritize public health and safety over commercialization, ensure that outpatient and inpatient treatment for youth is available on demand, and adopt family-centered approaches that recognize the impact of addictions on families and include them in treatment. Additionally, there is a need for increased research on reducing cannabis-related harms in youth and young adults, enhanced education about risks and harms, and dissemination of information on lower-risk cannabis use guidelines. Finally, harm reduction approaches developed for adult substance use often overlook the biopsychosocial, familial, spiritual, cultural, legal, and ethical issues, specific to youth and young adults [48]. Therefore, it is critical to develop youth-centered harm reduction approaches that recognize the developmental needs and vulnerabilities of individuals up to age 25, as well as the important role of families.

## Data Availability

The datasets used and/or analysed during the current study are available from the corresponding author on reasonable request.

## Abbreviations

CCSA: Canadian Centre on Substance Use and Addiction
CCS: Canadian Cannabis Survey
CPS: Canadian Paediatric Society
FAR: Families for Addiction Recovery
LRCUG: Lower Risk Cannabis Use Guidelines
LRSUG: Lower Risk Substance Use Guidelines
LRSUG-PSYCH: Lower Risk Substance Use Guidelines for Psychosis
P: Parent
RA: Research assistant
SP: Service provider
SGMY: Sexual and gender minority youth
THC: Tetrahydrocannabinol
Y: Youth
